# Encoding of Spatial Attention by Primate Prefrontal Cortex Neuronal Ensembles

**DOI:** 10.1523/ENEURO.0372-16.2017

**Published:** 2018-03-21

**Authors:** Theda Backen, Stefan Treue, Julio C. Martinez-Trujillo

**Affiliations:** 1 Department of Physiology, McGill University, Montreal, H3G 1Y6, Canada; 2 German Primate Center, Leibniz Institute for Primate Research, Goettingen, 37077, Germany; 3 Bernstein Center for Computational Neuroscience, Goettingen, 37073, Germany; 4 Robarts Research Institute, University of Western Ontario, London, N6A 5B7, Canada; 5 Department of Physiology & Pharmacology, University of Western Ontario, London, N6A 5B7, Canada; 6 Brain and Mind Institute, University of Western Ontario, London, N6A 5B7, Canada; 7 Department of Psychiatry, University of Western Ontario, London, N6A 5B7, Canada

**Keywords:** Decoding, neuronal ensembles, prefrontal cortex, primate, spatial attention

## Abstract

Single neurons in the primate lateral prefrontal cortex (LPFC) encode information about the allocation of visual attention and the features of visual stimuli. However, how this compares to the performance of neuronal ensembles at encoding the same information is poorly understood. Here, we recorded the responses of neuronal ensembles in the LPFC of two macaque monkeys while they performed a task that required attending to one of two moving random dot patterns positioned in different hemifields and ignoring the other pattern. We found single units selective for the location of the attended stimulus as well as for its motion direction. To determine the coding of both variables in the population of recorded units, we used a linear classifier and progressively built neuronal ensembles by iteratively adding units according to their individual performance (best single units), or by iteratively adding units based on their contribution to the ensemble performance (best ensemble). For both methods, ensembles of relatively small sizes (*n* < 60) yielded substantially higher decoding performance relative to individual single units. However, the decoder reached similar performance using fewer neurons with the best ensemble building method compared with the best single units method. Our results indicate that neuronal ensembles within the LPFC encode more information about the attended spatial and nonspatial features of visual stimuli than individual neurons. They further suggest that efficient coding of attention can be achieved by relatively small neuronal ensembles characterized by a certain relationship between signal and noise correlation structures.

## Significance Statement

Single neurons in the primate lateral prefrontal cortex (LPFC) are known to encode the spatial location of attended stimuli as well as other visual features. Here, we investigate how these single neuron coding properties translate into how ensembles of neurons encode information. Our results show that LPFC neuronal ensembles encode both the allocation of attention and the direction of motion of moving stimuli with higher efficiency than single units. Furthermore, relatively small ensembles reach the same decoding accuracy as the full ensembles. Our findings indicate that information coding by neuronal ensembles within the LPFC depends on complex network properties that cannot be solely estimated from coding properties of individual neurons.

## Introduction

Electrophysiological studies in nonhuman primates have shown that neural activity in the lateral prefrontal cortex (LPFC) is strongly modulated by selective visual attention: the responses of single neurons to attended targets are enhanced relative to responses to unattended distractors (Rainer et al., 1998; Buschman and Miller, 2007; Bichot et al., 2015). For the case of spatial attention, these phenomena translate into the encoding of attended relative to unattended locations by individual single neurons (Lennert and Martinez-Trujillo, 2011). However, several studies have suggested that information coding by neuronal populations cannot be determined simply from measurements in individual neurons. The amount of information encoded by a population of neurons also depends on interactions between simultaneously active neurons that generate correlated firing (Maynard et al., 1999; Averbeck et al., 2006; Seidemann et al., 2009). This is important to consider in neurophysiological studies, since behavior is likely generated by the activity of neuronal ensembles composed of many individual neurons sharing connectivity patterns rather than by the activity of single neurons acting in isolation.

Technological advances, such as the emergence of tetrodes and multielectrode recording arrays, allow progress beyond the single-neuron approach in electrophysiological studies toward investigating the properties of many neurons recorded simultaneously (neuronal ensembles; Yuste, 2015). Many studies in macaque monkeys using multielectrode array recordings have examined the encoding of signals in cortical areas related to motor processing to further the development of neural prosthetics (Wessberg et al., 2000; Musallam et al., 2004). However, relatively fewer studies have examined executive control signals related to attention in high-order associative areas using the same techniques (Kiani et al., 2015; Tremblay et al., 2015; Astrand et al., 2016). For the LPFC, it has been recently demonstrated that ensembles of neurons encode information about the allocation of spatial attention, and that the encoded information is influenced by correlated firing between units (i.e., noise correlations; Tremblay et al., 2015). One issue that remains less investigated is whether LPFC neuronal ensembles can encode other task-relevant (nonspatial) features of visual stimuli. Furthermore, it remains unclear how the coding of spatial locations and other features changes as a function of the size and composition of neuronal ensembles.

To address these issues, we simultaneously recorded the activity of neurons in the LPFCs of two rhesus macaque monkeys while they allocated attention to one of two moving random dot patterns (RDPs) positioned in opposite hemifields. The monkeys had to deploy spatial attention but then respond to a change in another feature of the stimulus: its motion direction. We find that individual units were tuned for both the attended location and the motion direction of the stimulus. However, there is little overlap between the coding of spatial attention and motion direction in the populations of recorded units. Using a linear classifier, we demonstrate that the performance of neuronal ensembles at decoding the attended location or the motion direction of the stimulus is higher than the performance of the best single units. Finally, we compare the performance of ensembles built using different methods and found that ensembles of relatively small sizes (<60), that do not necessarily include the best performing individual units, maximize decoding accuracy.

## Materials and Methods

### Animals

Two adult male monkeys (*Macaca mulatta*; R, 9.7 kg; S, 10.2 kg) participated in the experiments. All animal procedures were performed in accordance with the McGill University animal care committee regulations. During the training and testing periods, the animals received fluids as reward for correctly performing the task. We also gave the animals fresh fruits and vegetables as supplements when finishing a session. Body weight, water intake, and mental and physical well-being were monitored daily. None of the animals were killed for the purpose of this study.

### Visual stimuli

The stimuli were back-projected on a screen using a video projector (NEC WT610, 1024 × 768-pixel resolution, 75 Hz) and custom-made software running on an Apple G4 Power PC. The animals viewed the screen at a distance of 57 cm (i.e., 1 cm on the screen corresponded to 1° of visual angle). The stimuli were random dot patterns (RDPs) generated by plotting colored dots (white, 76.39 cd/m^2^; gray, 10.83 cd/m^2^; pink, 22.68 cd/m^2^; green, 11.26 cd/m^2^; blue, 10.96 cd/m^2^; red, 8.92 cd/m^2^; turquoise, 44.14 cd/m^2^) on a dark gray background (0.74 cd/m^2^) with a density of 3 dots/deg^2^ within a circular stationary virtual aperture. All dots within one RDP moved coherently at a speed of 15°/s and were replotted at the opposite side when they crossed the border of the aperture. The radius of the aperture was 4°, and it was centered 8° from the fixation spot.

### Task

The animals initiated a trial by keeping gaze within a 2°-radius window (4° in monkey S) centered on a small fixation spot (0.24 deg^2^). Gaze position was monitored using an infrared video-based eye tracker (EyeLink 1000, SR Research). After a 353-ms fixation period, two moving RDPs appeared, one located to the left and the other to the right of the fixation spot. The patterns were composed of white dots on a dark background that moved either up (0°) or down (180°) relative to the vertical. After a variable interval (294, 471, or 647 ms) after the RDPs’ onset, both patterns changed to different colors (i.e., the left one to green and the right one to red). The task for the animals was to identify one of the two RDPs as the target based on its color and covertly attend to it while ignoring the other (the distractor). After 706 ms, the color was removed and the RDPs returned to white. The animals had to maintain attention on the target and wait 753–1600 ms for a brief motion direction change in the target stimulus (118-ms duration, 32° clockwise from the current direction) and release the button within 100–650 ms. In 50% of the trials, the distractor changed motion direction before the target. In those trials, the monkey had to keep holding the lever until the target changed. Which of the two colors indicated a target was based on an ordinal color-rank rule the monkey had learned over the training sessions (turquoise > red > blue > green > pink > gray; Lennert and Martinez-Trujillo, 2011, 2013). Each correctly performed trial was rewarded with a drop of juice. A sequence of correct trials yielded a slight increase in reward size. Trials in which the monkey responded to the distractor change (false alarms), did not respond to the target change within the reaction time window (misses), or broke fixation before the end of a trial (fixation breaks), were terminated without reward. The different trial types were presented in random sequence. Only correctly performed trials were included in the analysis unless otherwise indicated.

### Surgical procedures

The surgical operations were conducted under general anesthesia using isofluorane administered through endotracheal intubation. The animals were implanted with titanium head posts used to restrain head motion during training and recording sessions. We chronically implanted a 10 × 10 multielectrode array (96 channels, Blackrock Microsystems) in each monkey’s left LPFC. The array was positioned on the cortical surface anterior to the knee of the arcuate sulcus and caudal to the posterior end of the principal sulcus, known as area 8A in the macaque monkey (Petrides, 2005).

### Electrophysiological recordings

We recorded from all 96 channels from the left LPFC of both animals. Data were recorded using a Cerebus Neuronal Signal Processor (Blackrock Microsystems) via a Cereport adapter. After 1× amplification in the head stage (ICS-96), the neuronal signal was bandpass filtered (0.3 Hz to 7.5 kHz) and digitized (16 bit) at a sample rate of 30 kHz. For each channel, spike waveforms were detected by manually thresholding (∼4 times the root mean square of the noise amplitude) the digitally high-pass filtered (250 Hz, 4 pole) raw voltage trace. The extracted spikes and associated waveforms were sorted offline using both manual and semiautomatic techniques using OfflineSorter (Plexon) and Matlab (MathWorks).

### Data analysis

Analysis of spike data (firing rates) and statistical tests were performed using Matlab. Unless indicated otherwise, our analyses were computed for a 500-ms window during a postcue/sustained attention period (150 ms after color offset; 650 ms after color offset). For further details on the results of the statistical analyses, refer to Table 1.

**Table 1.** Statistical analyses

Location	Comparison	Data Structure	Type of Test	Observed Power
a	Task-relatedness in monkey R and S	Normally distributed	Wilcoxon rank-sum	R: 2.89 × 10^−206^ – 0.0481; S: 3.79 × 10^−102^ – 0.0498
b	Single-unit selectivity in monkey R	Normally distributed	2-factor ANOVA	Location: 2.08 × 10^−58^ – 0.0486; Direction: 1.16 × 10^−12^ – 0.0496
	Single-unit selectivity in monkey S	Normally distributed	2-factor ANOVA	Location: 3.13 × 10^−5^ – 0.0496; Direction: 7.54 × 10^−38^ – 0.0445
c, f	Location selectivity vs. chance in monkey R (c) and S (f)	Normally distributed	χ^2^ test	R: 8.29 × 10^−10^; S: 0.6972
d, g	Direction selectivity vs. chance in monkey R (d) and S (g)	Normally distributed	χ^2^ test	R: 0.0019; S: 4.13 × 10^−10^
e, h	Overlapping selectivity vs. chance in monkey R (e) and S (h)	Normally distributed	χ^2^ test	R: 0.0040; S: 0.1016
i	Latency of significant difference in responses to preferred and nonpreferred location in monkey R and S	Normally distributed	Student’s *t* test	R: *T* = 12.36 ± 5.09; *p* = 2.46 × 10^−45^ – 0.0235; S: *T* = 4.15 ± 1.61; *p* = 2.98 × 10^−8^ – 0.0486
j	Latency of difference in responses to directions in monkey R and S	Normally distributed	Student’s *t* test	R: *T* = 7.14 ± 2.98; *p* = 4.63 × 10^−21^ – 0.0461; S: *T* = 11.41 ± 3.57; *p* = 4.79 × 10^−37^ – 0.0253
k, o	Decoding attended location using BSU approach vs. chance in monkey R (k) and S (o)	Normally distributed	Exact test	R: <0.01; S: >0.2400
l, *p*	Decoding attended location using BE approach vs. chance in monkey R (l) and S (*p*)	Normally distributed	Exact test	R: <0.01; S: >0.08
m, n	Decoding location from decorrelated BE (m) and BSU (n) ensembles in monkey R	Normally distributed	Paired *t* test	BE: *p* = 0.0091 – 0.0489; BSU: *p* = 0.2074
q, r	Decoding direction from decorrelated BE (q) and BSU (r) ensembles in monkey S	Normally distributed	Paired *t* test	BE: *p* = 1.59 × 10^−4^ – 0.0495; BSU: *p* = 0.0373 – 0.0499
s	Comparing BE Nmax decoding performance in monkey S to chance	Normally distributed	Exact test	0.8000
t, u	Decoding motion direction using BSU approach vs. chance in monkey R (t) and S (u)	Normally distributed	Exact test	R: <0.01; S: <0.01
v, w	Decoding motion direction using BE approach vs. chance in monkey R (v) and S (w)	Normally distributed	Exact test	R: <0.01; S: <0.01
x, y	Decoding location from decorrelated BE ensembles in monkey R (x) and S (y)	Normally distributed	Paired *t* test	R: *p* = 1.43x10^−4^ – 0.0469S: *p* = 6.19x10^−5^ – 0.0458
z, aa	Comparing location decoding between animals using BE (z) and BSU (aa)	Normally distributed	Unpaired *t* test	BE: 5.69 × 10^−7^ – 2.99 × 10^−4^; BSU: 1.05 × 10^−5^ – 4.02 × 10^−4^
bb, cc	Comparing motion direction decoding between animals using BE (bb) and BSU (cc)	Normally distributed	Unpaired *t* test	BE: 2.74 × 10^−6^ – 0.0080; BSU: 1.46 × 10^−5^ – 0.0191
dd, ee	Comparing max. location decoding performance between ensemble types in monkey R (dd) and S (ee)	Normally distributed	Wilcoxon signed-rank	R: 0.1250; S: 0.0625
ff, gg	Comparing max. direction decoding performance between ensemble types in monkey R (ff) and S (gg)	Normally distributed	Wilcoxon rank-sum	R: 0.1250; S: 0.0625
hh	Comparing maximum location decoding performance between ensemble types for each recording session	Normally distributed	Wilcoxon signed-rank	0.0039
ii	Comparing maximum direction decoding performance between ensemble types for each recording session	Normally distributed	Wilcoxon signed-rank	0.0039
jj	Comparing ensemble sizes with maximum decoding performance across stimulus features	Normally distributed	Wilcoxon signed-rank	0.0198
kk	Comparing ensemble sizes with 90% of maximum decoding performance across stimulus features	Normally distributed	Wilcoxon signed-rank	0.0293
ll, mm	Decoding attended location across all trial outcomes vs. chance in monkey R (ll) and S (mm)	Normally distributed	Exact test	R: <0.01; S: 0.2840
nn, oo	Decoding location across all hit trials vs. chance in monkey R (nn) and S (oo)	Normally distributed	Exact test	R: <0.01; S: 0.0160
pp, qq	Decoding location across all error trials vs. chance in monkey R (pp) and S (qq)	Normally distributed	Exact test	R: 0.4300; S: 0.3760
rr, ss	Decoding location across all false positive trials vs. chance in monkey R (rr) and S (ss)	Normally distributed	Exact test	R: 0.4600; S: 0.6600
tt, uu	Decoding motion direction in across all outcomes vs. chance in monkey R (tt) and S (uu)	Normally distributed	Exact test	R: <0.01; S: <0.01
vv ww	Decoding motion direction across all hit trials vs. chance in monkey R (vv) and S (ww)	Normally distributed	Exact test	R: <0.01; S: <0.01
xx, yy	Decoding motion direction across all error trials vs. chance in monkey R (xx) and S (yy)	Normally distributed	Exact test	R: 0.5500; S: <0.01
zz, aaa	Decoding motion direction in false positive trials vs. chance in monkey R (zz) and S (aaa)	Normally distributed	Exact test	R: 0.3850; S: <0.01

Superscript letters refer to the statistical tests in figures, Results, and Tables 2 and 3.

From the recordings, we extracted a total of 1081 units [556 in monkey S over 5 sessions (156, 103, 107, 102, 88) and 525 in monkey R over 4 sessions (151, 107, 127, 140)] with a firing rate higher than 0.1 Hz. We used a Wilcoxon rank-sum test to determine whether units had significantly different (*p* < 0.05) firing rates in the postcue period or in a 500-ms window during the color cue presentation compared with baseline, which was a 700-ms window centered around stimulus onset. This resulted in 391 units in monkey S (70%) and 462 units in monkey R (88%).

#### Neuronal selectivity

We determined neuronal selectivity by performing two-way ANOVA using the factors target location and motion direction. For units with a significant main effect, we determined which of the stimulus parameters yielded the highest mean firing rate (i.e., a unit that showed a main effect of location was considered ipsi-selective when its mean firing rate was higher for targets presented ipsilaterally to the recording site than for contralaterally presented targets).

#### Spike density functions

The activity of single selective units and the selective populations were plotted as trial-average spike density functions, generated by convolving the spike train with a Gaussian kernel (width 25 ms) and normalizing by the maximum firing rate in each unit’s preferred stimulus condition. We determined the latency and magnitude of the difference in response to preferred and nonpreferred features by comparing the mean activity of the selective units in 20-ms bins using a paired *t* test (*p* < 0.05). Once five consecutive bins were significant, we took the first of those as the latency of the selectivity. We used the mean *t*-value across all 20-ms bins to quantify the magnitude of the difference.

#### Significant proportions of selective units

We investigated whether our proportions of selective units found in each animal were significantly different from chance by determining the proportions in a randomized population. For this, we randomly permuted the entire trial order and recomputed the ANOVA. We repeated this permutation and recomputation 1000 times, compared the mean proportions of the shuffled populations to the actual proportions in the data using a χ^2^ test (*p* < 0.05), and calculated 95% confidence intervals using the Wilson score interval.

#### Anatomic clustering

We determined whether location- and direction-selective units were significantly organized or clustered in space across the array using Moran’s *I*, a metric of spatial autocorrelation (Moran, 1950). Moran’s *I* ranges from –1 to 1, with negative values indicating that similar feature values are spatially repellant and positive values indicating that similar values are spatially clustered. We compared our values to chance, obtained by shuffling each electrode’s feature label 1000 times and then taking the 95th percentile range of values.

#### Decoding stimulus features

We used a L2-regularized linear support vector machine (SVM, liblinear v2.1; Fan et al., 2008) to decode the stimuli features (specifically, target location and motion direction) from the task-related units during the postcue epoch when both RDPs had the same color and direction. The regularization parameter was the optimal penalty parameter *C* (refer to Eq. 1 in Fan et al., 2008), identified by conducting a grid search. To assess the accuracy of the decoder, we used a cross-validation technique: The decoder was trained on 90% of the trials for a given neural ensemble and then tested on the remaining 10% (10-fold cross-validation). The SVM was iteratively trained and tested on different subsamples of the trials until each trial was in the test set at least once and used for training nine times. Furthermore, we balanced the number of trials between the conditions by identifying the minimum number of trials for the unique conditions. Of the other conditions, a random subsample equal to the minimum number of trials was selected. We repeated the subsampling procedure 10 times.

We used two different procedures to determine decoding performance for neuronal ensembles of various sizes. First, we sorted the units from highest to lowest by their individual performance in decoding attended location and motion direction, respectively. To build an ensemble of size *n* + 1, we iteratively added the next-best-performing unit to the ensemble of size *n*, i.e., the highest performing unit was considered an ensemble of size 1, and to build the *n* + 1 ensemble we added the second-best performing unit, and so forth to build increasingly larger ensembles. We refer to the resulting ensemble from this method as the best single-unit ensemble (BSU). We also used a second procedure, best ensemble (BE), by again starting with the best single unit but then paired it with all remaining units to find the pair that maximized the decoder’s performance. Then we used this pair and combined it iteratively with each of the remaining units to find the best trio that maximized the decoder’s performance. The procedure was repeated for the best quartet and so on. Note that the BE method optimizes ensemble performance and allows for the possibility that the best single units do not necessarily make up the best ensembles. Because we recorded a different number of units each day, we performed this analysis only for the minimum ensemble size across all recording sessions for each animal (94 for monkey R and 61 for monkey S). We used unpaired *t* tests (*p* < 0.05) to compare the decoder’s performances for the different ensemble types between monkeys. To assess the contribution of correlations in the ensemble, we shuffled trial order within the same condition to destroy shared trial-by-trial variability and recomputed the SVM classification analysis on those shuffled ensembles. This procedure was repeated 100 times. We used paired *t* tests (*p* < 0.05) to assess differences in decoding accuracy with and without correlations. Furthermore, we used exact tests to compare the obtained decoding performance at each ensemble size to chance performance, which we obtained by randomizing the entire trial order and rebuilding the ensembles for each surrogate data set. We repeated this shuffling procedure 10 times. Last, we compared the maximum decoding performance and the ensemble size at which it was reached with Wilcoxon signed-rank tests (*p* < 0.05). Superscript letters listed with *p*-values correspond to the statistical tests shown in Table 1.

#### Decoding behavior

We predicted target location and motion direction from the activity of the BE yielding the maximal decoder performance under various behavioral conditions. Specifically, we compared decoding accuracy between all trials (1) considering correct trials only, (2) considering error trials only, and (3) narrowing included error trials down to false positives (i.e., responses to the distractor) only. Because of only a small number of error and false-positive trials, we were unable to balance the number of trials between the different outcome conditions. Thus, we restricted statistical analyses to use exact tests (*p* < 0.05) for comparing the decoding performance and chance performance, obtained by shuffling trial labels 100 times and recomputing the decoding accuracy.

## Results

### Behavioral performance

**Figure 1.**
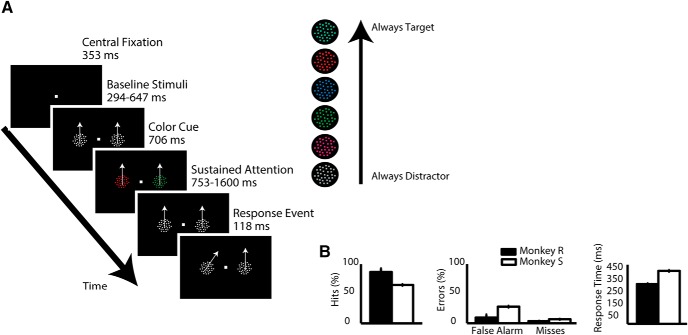
Behavioral task and performance. ***A***, Example trial of color scale task and hierarchy of colors used (inset). The monkeys initiated a trial by fixating on the central point while pressing a button. After this initial fixation period, two white moving RDPs appeared peripherally of the fixation point and changed to two different colors after a random interval. The animals had to identify the higher-ranking color (the target) and allocate their attention to it before the color cue was extinguished and the RDPs returned to white. The monkeys had to maintain central fixation and covert attention until there was a brief motion direction change in the relevant stimulus. In 50% of the trials, the distractor changed before the target, in those cases, the monkeys had to keep pushing the button as only a release after the target change was rewarded with juice. ***B***, Percentage of hits, errors, and mean response time for monkey R (black bars) and monkey S (white bars). Averaged across all color combinations. Error bars denote standard deviation across sessions.

We trained two adult monkeys (*Macaca mulatta*), R and S, to maintain their gaze on a central fixation point while covertly attending to one of two white, peripherally presented, moving RDPs presented on a dark background. In a given trial, the RDPs appeared simultaneously and moved in the same direction, changing colors after a variable time. Based on a color rule (Lennert and Martinez-Trujillo, 2011, 2013), the monkeys had to identify and attend to the target RDP while ignoring the other RDP (distractor). Briefly, we taught the animals an arbitrarily arranged ordinal hierarchy of six isoluminant colors. In each trial, the higher-ranking colored RDP was the target. The animals were rewarded for releasing a button after correctly detecting a brief motion direction change in the target, ignoring distractor changes (Fig. 1*A* and Methods).

Both monkeys learned the task and performed above chance level (50%) in all experimental sessions (Fig. 1*B*, left panel; 87.6% correct trials in monkey R and 64.97% correct trials in monkey S, respectively). Most error trials of both monkeys were responses to the distractor (false alarms) rather than failures to respond (misses; Fig. 1*B*, middle panel). The latter indicates that the animals indeed attended to the target and ignored the distractor but also that the task was challenging for the animals. Monkey R had a better performance and faster reaction times than monkey S (Fig. 1*B*, right panel, 327 and 436 ms, respectively). Unless stated otherwise, we considered only correct trials for our analyses of neuronal responses.

### Neuronal selectivity

**Figure 2.**
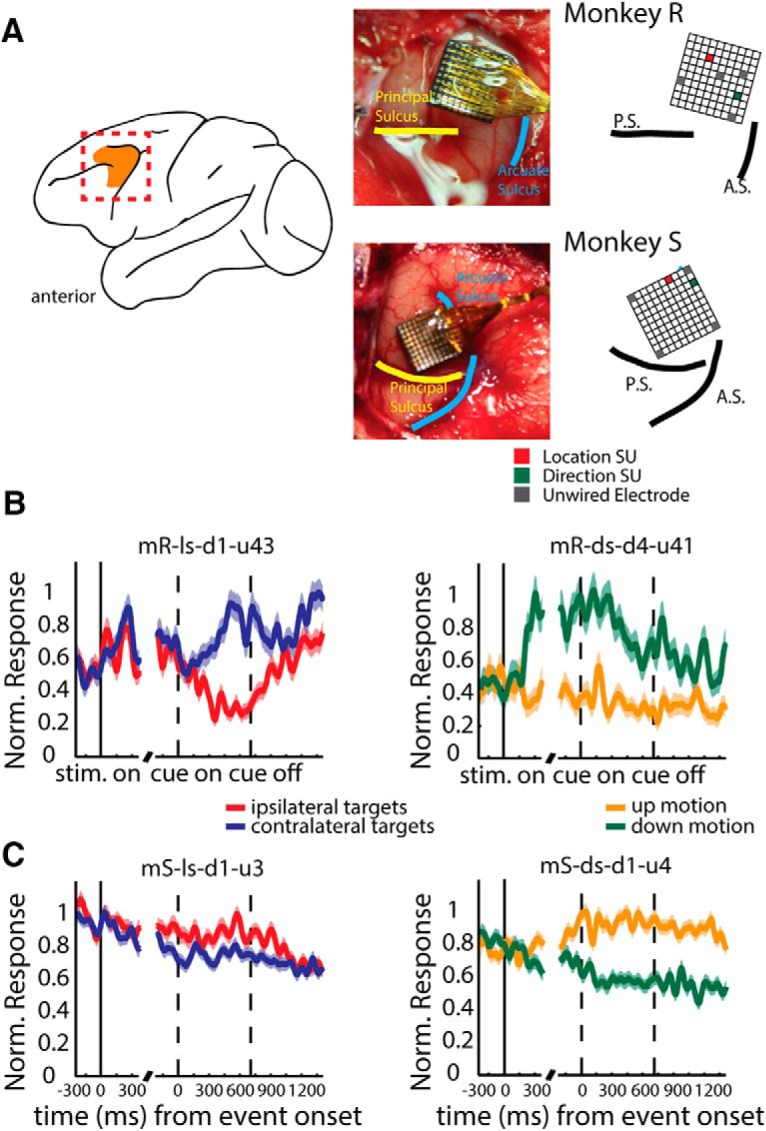
Implantation sites of the arrays and single-unit activity. ***A***, Schematic macaque brain with area 8 a highlighted according to Petrides (2005) and implantation sites. Photographs were taken during the implantation procedure. Principal and arcuate sulci are indicated. ***B***, Single-cell examples obtained from monkey R illustrating mean normalized responses (ordinate) to different stimulus conditions as a function of time from stimulus onset (left abscissa) and color change onset (right abscissa). Schematic shows the position of the units on the array. Prominent landmarks are indicated. ***C***, Single-cell examples obtained from monkey S for the same conditions. Shading represents SEM (±) at each time point.

While the animals performed the task, we recorded the responses of a total of 556 units (single units and multiunits) in monkey S (5 sessions) and 525 in monkey R (4 sessions) using 96-channel microelectrode (Utah) arrays chronically implanted in the left area 8A, located on the cortical surface between the posterior end of the principal sulcus and the knee of the arcuate sulcus (Petrides, 2005). Our arrays were located slightly dorsal to the principal sulcus (Fig. 2*A*). Of those recorded, 853 units (79%) showed significantly different firing rates during the color cue presentation and/or the postcue epoch compared with a window of 700 ms centered at stimulus onset (Wilcoxon rank-sum test; *p* < 0.05^a^). The variables of interest in the following analyses were the allocation of spatial attention and the direction of the stimuli; therefore, we concentrated on the postcue or attentional period in which the stimuli on the screen did not change color or direction.

To examine the tuning of single units, we used a 2-way ANOVA with target location and motion direction as factors (*p* < 0.05^b^). Fig. 2*B* shows an example unit (recorded from monkey R) that responded more strongly when the target location was contralateral to the recording site (left panel), or when the RDP’s motion direction was down (right panel). Similarly, Fig. 2*C* shows an example unit (recorded from monkey S) responding more strongly on trials when the target stimulus was presented ipsilaterally (left panel), or when the RDPs’ motion direction was up (right panel). These units were recorded from different electrodes in the array (see schematics in Fig. 2*A*), and they encode the target location (red squares) and motion direction (green squares), respectively.

**Figure 3.**
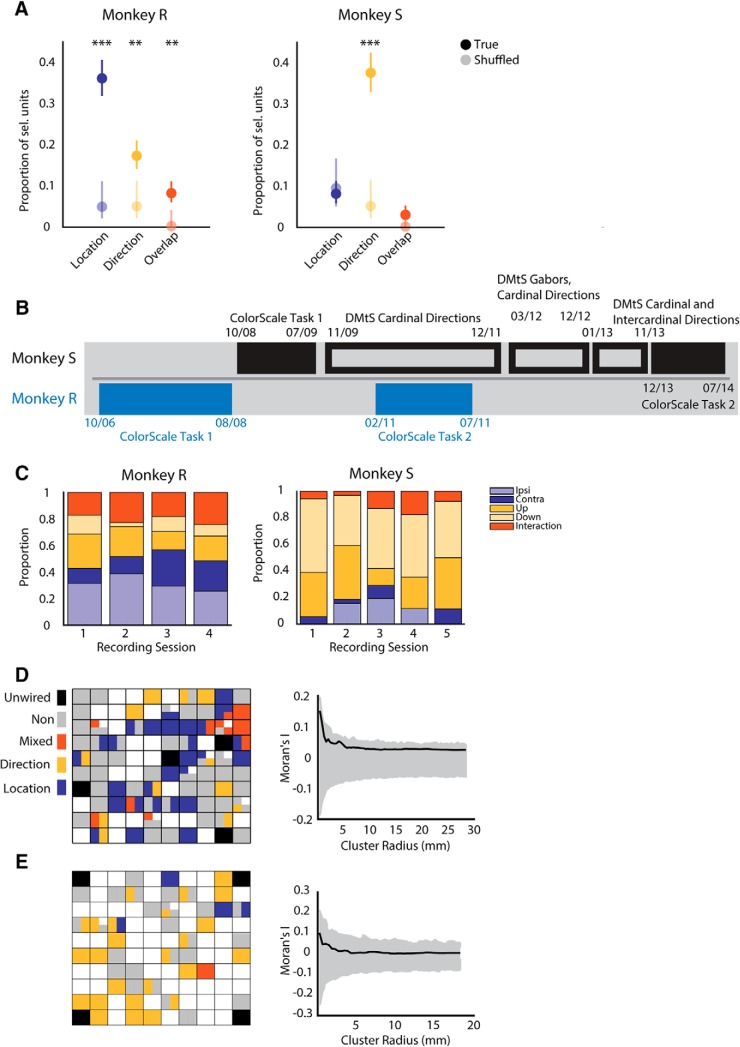
Proportions of selective single units. ***A***, Proportions of selective units were obtained using two-way ANOVA with the factors target location and motion direction in monkey R (right) and monkey S (right) during the postcue period. The markers represent the proportions of selective units found in the population (blue, location; yellow, direction; orange, selectivity for both); the error bars represent 95% confidence intervals. Shading indicates proportion found in data with shuffled trial labels. Asterisks mark significant differences in proportions compared to chance proportions (***, *p* < 0.001; **, *p* < 0.05, χ^2^-test). The majority of selective cells found in monkey R were location selective, and the majority of selective cells found in monkey S were direction selective. ***B***, Timeline of the monkeys’ training. At the time of recording the task presented in this paper (ColorScale Task 2), monkey R had received exclusive training on a spatial attention task involving a color scale (Lennert and Martinez-Trujillo, 2011; 2013). Despite a 2.5-yr pause between the two tasks, monkey R performed the task very well. After its initial training, monkey S was extensively trained on delayed-match-to-sample tasks involving motion directions (for example, Mendoza-Halliday et al., 2014). Monkey S had a >4-yr break from a color scale task, during which it became an expert for motion direction tasks. ***C***, We tracked the proportion of selective electrodes/channels per recording session in each animal to see whether the distributions were approximately stable over time. To test the spatial clustering hypothesis, each electrode’s categorical selectivity of on example session was mapped into the array for monkey R (***D***) and monkey S (***E***). Left panels: colors indicate whether units on an electrode were selective. White channels had no activity; black channels indicate unwired electrodes. Right panels: magnitude of spatial clustering of preferred stimuli in monkey R (***D***) and S (***E***). Black line depicts Moran’s I (metric of spatial autocorrelation) calculated over increasing spatial scales. Gray shaded area represents chance values.

We next examined the proportions of selectivity for the attended location (location selective) and the stimulus’ motion direction (direction selective) in the entire population (Fig. 3*A*). In monkey R (left panel), we identified 56% of the units (285 of 462) to be selective for at least one of the two variables, attended location and motion direction. Of those, 59% (36% ± 4.38% of the total population) were location selective, 28% (17% ± 3.45%) were direction selective, and 13% (8% ± 2.52%) showed selectivity for both attended location and direction. To determine whether these proportions were different from those expected by chance, we compared them to those obtained using a randomization procedure (chance estimate). For the randomization procedure, we used the same trials and units as in the original data but shuffled the trial labels. In monkey R, the proportion of location selective cells predicted by chance was 4.94%, which was significantly smaller than that found in the real population (χ^2^ test, *p* = 8.29 ×10^−10 c^). Similarly, the proportion of direction-selective units was significantly smaller in the randomized population than in the real data (5.04%, χ^2^ test, *p* = 0.002^d^), as well as the proportion of units encoding both variables (0.23%, χ^2^ test, *p* = 0.004^e^).

In monkey S (Fig. 3*A*, right panel), 49% of the units (191 of 391) were classified as selective for the attended location or direction. The majority of cells, 77% (38% ± 4.8% of the total population), were direction selective, 17% (8% ± 2.75%) were location selective, and 6% (3% ± 1.71%) were both location and direction selective. The proportion of direction-selective units was significantly higher than expected by chance (5.21%, χ^2^ test, *p* = 4.13 ×10^−10 g^), whereas the proportion of location selective units was not significantly different from that found in a randomized population (9.39%, χ^2^ test, *p* = 0.6972^f^). The proportion of units selective for both features was also not different from chance (0.21%, χ^2^ test, *p* = 0.1016^h^). To assess whether the (nonsignificant) proportion of units that did have location selectivity showed a true effect, we compared the size of the isolated effect in the original data with that in the shuffled data by computing an index of sensitivity (*D*′). The magnitude of the effect was similar in both groups of units (Wilcoxon rank-sum test, *p* = 0.553). Thus, it is unclear whether the effect isolated in the original data was a true effect of attention that is present in a small number of units or reflected noise in our data. With the current analysis, we cannot fully reject the latter scenario.

The distributions of selectivities were different in the two animals. Whereas animal R had a larger proportion of units selective for the attended location than units selective for motion direction, animal S showed an inverse pattern. One possible explanation for this result is that the animals had different training histories and because selectivity in the LPFC for different features may be affected by experience; thus exposure to different tasks may have shaped neuronal selectivities in a different manner for each animal. To investigate this issue, we plotted the training history of the animals (Fig. 3*B*). Monkey R was extensively trained and participated in other experiments using a similar color-rank order task shown in Fig. 1. In this task, although motion direction is important to detect the response cue, the spatial location of the target is of primary importance, i.e., the animals had to decide whether the right or left RDP was the target. On the other hand, monkey S had first been trained in the same task, but before undergoing testing in the current experiments, it was extensively trained in various match-to-sample tasks that required matching the direction of two moving RDPs or Gabor patches. In those tasks, location was an irrelevant variable for determining what the target was: only motion direction was important (Mendoza-Halliday et al., 2014). Using an ANOVA with the factors target location and motion direction on the averaged activity recorded on each electrode, we tracked the distribution of selective channels over the recording sessions in each animal (Fig. 3*C*). Our goal was to examine whether the proportions of selective cells were relatively stable over time. Interestingly, from one session to the next, there were only few channels with significant tuning in common, but overall there were very similar distributions of selectivities (see color bars). This suggests that the selectivity was stable over recording time and the difference between animals was not due to an outlier session. Thus, it is possible that the differences in neuronal selectivities are due to differences in training history between animals. However, another equally possible explanation is that the area we recorded from was slightly different in both animals and the proportion of units may change depending on the relative location of the arrays. To test this hypothesis, we wanted to assess whether there was significant spatial clustering and/or even a difference therein. We mapped the preferred location or motion direction of an electrode onto its cortical position (the left panels in Fig. 3*D*,*E* show representative example sessions for each monkey). To examine whether neurons with similar preferences were anatomically clustered (Fig. 3*D*,*F*, right panels), we used Moran’s *I*, a metric of spatial autocorrelation, and compared it to the 95th percentile range of chance values obtained by shuffling the electrodes’ preference labels 1000 times. Although some neurons with similar preferences were isolated from nearby electrodes, in general the analysis revealed no significant clustering neurons selective for attended location or stimulus motion direction in the areas covered by the arrays in the two monkeys. The lack of spatial clustering as well as the similar position (i.e., position of the arrays relative to the sulci) led us to favor the training history hypothesis to explain the differences in neuronal selectivity between animals.

**Figure 4.**
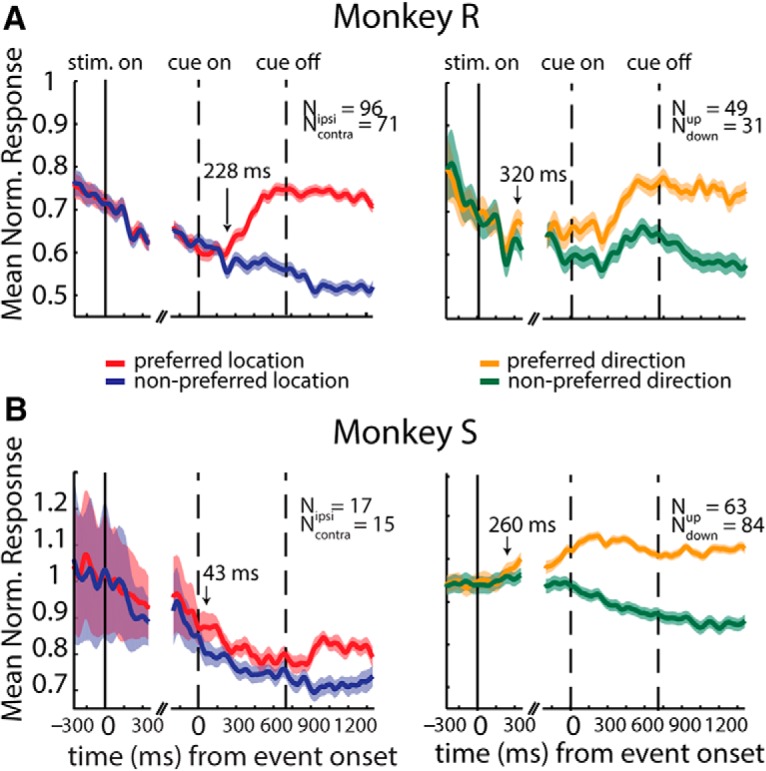
Population selectivities. Mean normalized population responses (ordinate) as a function of time from trial event onsets (abscissas) in monkey R (***A***) and monkey S (***B***). Left: the population of location-selective cells (*n* = 167 and *n* = 32) shows an increased response when the target is in the preferred location (red) after color cue onset (left dashed line) compared with trials in which the target is in the nonpreferred location (blue). Right: the population of direction selective cells (*n* = 80 and *n* = 147) shows an increased response in trials with the preferred motion direction (orange) compared with the response in trials with the nonpreferred direction (green). Shading represents SEM (±) at each time point. Arrows indicate the onset of separability between the two curves, determined as the time point when the data in at least five consecutive bins of 20 ms were significantly different from each other (paired *t* test, *p* < 0.05).

To examine the population activity profiles, we pooled the responses of units selective for the attended location and motion direction. Because units could be selective for one location (i.e., ipsilateral or contralateral) or motion direction (i.e., up or down), we pooled units after aligning their responses to their preferred direction or location. To gauge the latency and magnitude of the difference in response to preferred and nonpreferred stimuli, we performed a paired *t* test on the responses of the selective units using time bins of 20 ms. The latency was determined as the first of five consecutive significant bins (*p* < 0.05) and the magnitude as the mean *t*-value across all bins. The difference between responses to the preferred (red) and nonpreferred (blue) target location is more pronounced in monkey R than in monkey S (*t* = 12.36 ± 5.09^i^ and 4.15 ± 1.61,^i^ respectively; Fig. 4*A*,*B*, left panels). On the other hand, the difference in the responses to the preferred (orange) and nonpreferred (green) direction seems to be less distinct between the two animals (*t* = 7.14 ± 2.98^j^ and 11.41 ± 3.57^j^ for monkey R and S, respectively; Fig. 4*A*,*B*, right panels). One detail in this figure is that in the two animals, the discrimination between motion directions (time when the responses to the preferred and nonpreferred directions diverge; Fig. 4*A*,*B*, left panels, black arrow; 320 and 260 ms after stimulus onset for monkey R and S, respectively) appears to start earlier than the discrimination between the attended and unattended locations (time where responses to attended and unattended locations start diverging; Fig. 4*A*,*B*, right panels, black arrow; 228 and 48 ms after color cue onset in monkey R and S, respectively). This is likely because information about the stimulus direction was available to the animal earlier than information about the target location. In other words, the animals probably identified the motion direction earlier and then directed their attention to the target and ignored the distractor.

These results indicate that average population responses are modulated by attended location and the stimulus motion direction in both animals. However, the degree to which the populations do so is different between the animals, particularly for the case of spatial attention. This is also concordant with the higher behavioral performance in monkey R relative to monkey S.

### Decoding attended location and motion direction from neuronal ensembles

We used a binary linear classifier, support vector machine (Cortes and Vapnik, 1995; SVM), to decode spatial attention and the stimuli’s motion direction independently from ensembles of simultaneously recorded task-related units in each session. We used the SVM as a proxy to assess the ability of a downstream entity (single neuron or neuronal ensemble) to read out the information from the recorded LPFC neuronal ensemble. Decoding accuracy was assessed using a cross-validation procedure in which 90% of the data were used for training and the remaining 10% for testing (10-fold cross-validation; see Methods).

**Figure 5.**
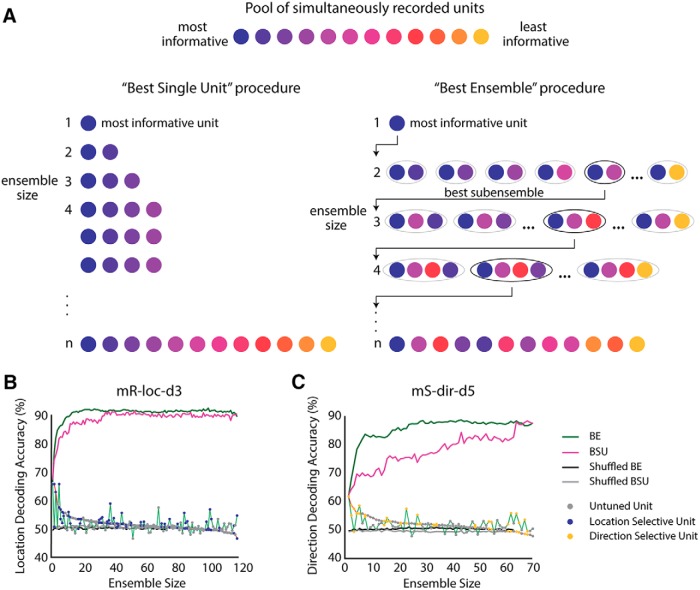
Ensemble building procedure. ***A***, We ranked individual units based on their information content, as assessed by SVM and then, starting with the most informative unit, either iteratively added the next best unit to the ensemble (BSU procedure) or looped through the remaining units to identify which pair yielded the highest performance, then looped through the remaining units to identify the best trio, etc. (BE procedure). ***B***, Example session from monkey R when decoding target location. The decoding accuracy in percentage is shown as a function of ensemble size for both building procedures (green, BE; magenta, BSU). Decoding accuracy expected by chance is shown in gray for BSU and in black for BE. Circular markers indicate the individual units’ decoding accuracy and the order in which they get added to the ensemble. Colored markers mark selectivity for the decoded feature. The red line connects the markers that make up the BSU ensemble, and the green line connects those that make up the BE ensemble. ***C***, Example session from monkey S when decoding motion direction.

The amount of information encoded by a neuronal ensemble has been shown to vary with the number of units in the ensemble (Tremblay et al., 2015). To investigate this issue, we used two different procedures of progressively building (adding units to) neuronal ensembles and obtained a decoder performance value for each ensemble size and composition. First, we decoded from each single unit independently and sorted the units based on their performance from highest to lowest. Then, we built neuronal ensembles (e.g., *n* = 2, 3, 4, … 94) by iteratively adding the next-best-performing unit (Fig. 5*A*, left, best single unit ensemble or BSU method). Second, we used a variation of this method: in each iteration, instead of adding the next-best-performing single unit to the ensemble, we added the unit that maximized the decoder’s performance when added to the ensemble. In this procedure, the existing ensemble of size *n* is paired with each one of the remaining units that have not been added, and a value of decoding accuracy for all *n* + 1 ensembles is obtained. The *n* + 1 ensemble that yielded the highest decoding was chosen. Then we kept this best-performing ensemble of *n* + 1 units and repeated the procedure (Fig. 5A, right). Note that applying this procedure does not search for the entire space of possible *n*-size ensembles, which was computationally unreachable in reasonable time. We refer to this building method as the best ensemble (BE; see also Methods).

The difference between these two procedures is that in the BSU procedure, performance of individual units dictates which unit is added to the ensemble. If the main factor that determines performance is the coding properties of individual units, this procedure should yield the best decoding performance with fewer units. On the other hand, in the BE procedure, the contribution of a unit to information coding by the entire ensemble determines its ensemble membership. In other words, the BE procedure takes into account not only the performance of the added unit considered in isolation but how the added unit interacts with the rest of the ensemble. If the tuning properties of individual units, and not the interactions, solely determine the ensemble performance, these two procedures should lead to identical ensembles of *n* units in each iteration (for all ensemble sizes).

Fig. 5*B*,*C* shows the results of two example sessions when decoding attended location in monkey R and motion direction in monkey S, respectively. The green lines indicate the decoding performance using the BE building method as a function of ensemble size, and the magenta lines indicate it for the BSU building method. The black and gray lines denote chance levels, obtained by shuffling the trial labels, for the BE and BSU ensembles, respectively. The circular markers show the individual units’ decoding performances in the order in which they were added to the ensembles. Colored markers indicate whether a unit was selective for either a target (attended) location (blue) or motion direction (yellow). For the BSU building method, we see, as anticipated, a steady decline in individual units’ decoding performances (red lines), whereas for the BE method, sometimes low performing/untuned units were added before high performing/tuned ones (green lines). Importantly, both ensemble methods consistently yield higher decoding accuracy than the best single-unit decoding accuracy (first unit on the *x*-axis or ensemble with *n* = 1). Note that the lines converge at the maximum ensemble size: for this *n*, the ensembles are the same and hence the decoding accuracy should be similar.

**Figure 6.**
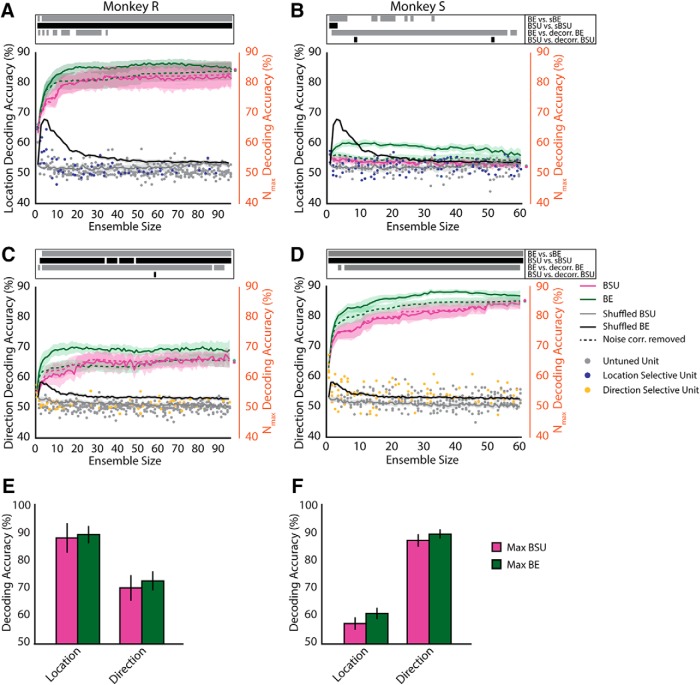
Decoding from neuronal ensembles using SVM. We decoded target location (***A***, ***B***) and motion direction (***C***, ***D***) during the postcue epoch from monkey R and S, respectively. The SVM’s performance (left ordinate) is shown as a function of ensemble size (abscissa). We truncated the plots to show only the performance for the minimum number of units across sessions. Green lines indicate decoding from BE ensembles, and magenta indicates BSU ensembles. Average decoding performance from ensembles built out of shuffled data are shown in black (BE) and gray (BSU). Dashed lines represent decoding from BE and BSE ensembles when noise correlations had been removed by shuffling trials within the same condition. Shading over the lines indicates SEM (±) for each ensemble size. The lines in the table on top indicate which ensemble sizes were significantly different from each other (*p* < 0.05) for the indicated comparisons. Circular markers indicate the individual units’ decoding performance once they got added to the ensembles. The right ordinate indicates decoding performance for the maximum ensemble sizes averaged across sessions. We compared median SVM performance of the ensembles that had produced the highest decoding accuracy for each stimulus class independently in monkey R (***E***) and monkey S (***F***). Error bars represent standard deviations across recording sessions.

Next, we confirmed these results at the population level (Fig. 6). Because the number of units we recorded varied between sessions, we considered the minimum ensemble size for our analyses (*n* = 94 in monkey R and *n* = 61 in monkey S), truncated the data accordingly, and plotted the average across sessions. We compared the decoder performance of the different ensemble types (magenta for BSU and green for BE) with the average performances based on surrogate data in which the trial labels were randomized 10 times and new ensembles for each shuffle were built (gray for shuffled BSU and black for shuffled BE) and with ensembles for which the trial labels had been permutated within condition 100 times, i.e., removing the simultaneity of recordings and effectively removing noise correlations (dashed lines). The lines inside the table on top of the plots indicate ensemble sizes that were significantly different from each other for the different comparisons (BE vs. shuffled, BSU vs. shuffled, BE vs. decorrelated BE, and BSU vs. decorrelated BSU).

When we decoded target location, the performance of all BSU sizes for monkey R (Fig. 6*A*) was significantly higher than chance performance (exact test, *p* < 0.01^k^). Similarly, the performance of 93 of 94 BE ensemble sizes was also higher than chance (exact test, *p* < 0.01^l^). Removing noise correlations from the ensembles reduced decoding accuracy for smaller ensembles (*n* < 40) using the BE method (paired *t* test, 0.0091 < *p* < 0.0489^m^) and had no effect on BSU ensembles (*p* > 0.2074^n^). For monkey S (Fig. 6*B*), we found the same trend across all BE ensemble sizes, although the exact tests failed to reach statistical significance (*p* > 0.08^p^). The same was true for the BSU ensembles (*p* > 0.2400^o^). Removing noise correlations had similar effects as in monkey R: it decreased decoding performance in the majority of BE ensembles (57/61, paired *t* test, 1.59 × 10^−4^ < *p* < 0.0495^q^) and only in few BSU ensembles (2/61, 0.0373 < *p* < 0.0499^r^). One reason we did not see a statistically significant difference between the data-based ensembles and those built on random data may be that the ensembles had different compositions that might have affect the comparison negatively. Nonetheless, our data indicate that even in monkey S, there is a systematic trend that relatively small and heterogeneous neuronal ensembles can encode stimulus information, even when the proportion of single units tuned for that feature in the ensemble is low. This may be due to the linear classifier weighing the contribution of different neurons to information coding unequally, as well as to the ability of the classifier to use information about the correlation structure of the neuronal ensemble. Note that the curve corresponding to the BE method in monkey S (green) reaches a maximum and then decreases as more neurons are added. This is likely because of the finite number of trials in our sample and the relatively low selectivity of neurons for the attended location. A small number of trials yields a too-high feature-to-instance ratio (i.e., neuron-to-trial). This leads to overfitting and consequently a decay in mean decoding accuracy (Trunk, 1979; Guyon and Elisseeff, 2003; Kanitscheider et al., 2015). In fact, the average decoding performance for the maximum possible BE ensemble size per recording session is not significantly different from chance (Fig. 6*B*, right ordinate, exact test, *p* = 0.8000^s^).

When examining decoding of motion direction, almost all BSU ensemble sizes for monkey R were significantly above chance (Fig. 6*C*, 89/94, exact test, *p* < 0.01^t^), and all ensemble sizes in monkey S were (Fig. 6*D*, exact test, *p* < 0.01).^u^ The BE also yielded significantly above-chance performances across 91 of 94 ensemble sizes in monkey R and across 60 of 61 ensemble sizes in monkey S (exact tests, *p* < 0.01^v^ and *p* < 0.01,^w^ respectively). Removing noise correlations again had a decreasing effect on most of the BE ensembles in both animals (paired *t* test, 1.43 × 10^−4^ < *p* < 0.0469^x^ and 6.19 × 10^−5^ < *p* < 0.0458^y^ for monkeys R and S, respectively) and yielded mostly no change in the BSU ensembles.

When decoding attended location using the BE method, we found higher decoding accuracy across all ensemble sizes in monkey R than in monkey S (unpaired *t* test, 5.69 ×10^−7^ ≤ *p* ≤ 2.99 ×10^−4 z^). In contrast, when decoding motion direction using the BE, performance was better in monkey S relative to monkey R (unpaired *t* test, 2.74 ×10^−6^ ≤ *p* ≤ 0.0080^bb^). The same was true when examining the BSU ensembles (unpaired *t* test, 1.05 ×10^−5^ ≤ *p* ≤ 4.02 ×10^−4 aa^ for location decoding and 1.46 ×10^−5^ ≤ *p* ≤ 0.0191^cc^ for direction decoding). These results follow the same trend as the differences in proportions of selective cells between animals (Fig. 3*A*). Notably, we were able to decode more information from either ensemble type than from the best single units (dots in all panels). This corroborates that ensembles encode substantially more information than the best single units and therefore than any measurement derived from statistics based on single-unit performance (e.g., average, median, or maximum performance across single units).

To more closely examine the differences in decoding performance between the BSU and BE ensemble-building methods, we assessed the maximum performances linked to each ensemble building method for both features (see Tables 2 and 3). It is important to note that in this analysis, the maximum performance is not necessarily equivalent to the performance of the full ensemble (see right ordinate axis in Figure 6*A–D*). Here, we defined maximum decoding performance as the average maximum decoding accuracy across recording sessions in the plotted ensemble sizes. The full ensemble does not necessarily yield the highest decoding performance, because our training/testing set has a finite number of trials. As mentioned earlier, training on finite data can lead to overfitting and suboptimal decoding accuracy (Trunk, 1979; Guyon and Elisseeff, 2003; Kanitscheider et al., 2015). The maximum performance can be considered as a low boundary estimate in the information encoded by the neuronal ensemble we recorded from.

**Table 2.** Ensemble decoding performance and ensemble size when decoding target location for each recording session in both animals

Recording session	Maximum BE performance (%)	BE size at maximum performance	BE size at 90% of maximum performance	Maximum BSU performance (%)	BSU size at maximum performance	BSU size at 90% of maximum performance	Total *n*
Monkey R							
Day 1	86.67	55	8	80.00	103	3	116
Day 2	87.55	52	6	86.12	27	11	94
Day 3	90.83	98	3	89.88	52	7	116
Day 4	93.34	69	4	92.01	122	8	136
Monkey S							
Day 1	60.69	11	1	55.65	30	47	112
Day 2	60.93	4	59	59.48	5	61	73
Day 3	63.41	12	1	59.30	70	31	77
Day 4	61.57	15	48	57.32	11	57	61
Day 5	57.73	15	61	54.30	1	55	68

Detailed list of what the maximum decoding accuracy was and at which ensemble size it was achieved, measured separately for the BE and BSU methods. Because the estimates are noisy and the decoding performance saturates, the ensemble sizes at which 90% of the maximum performance were achieved are also listed. The data are divided up into the individual sessions recorded from each animal. Decoding performances between ensemble types as well as ensemble sizes were compared across the nine sessions using Wilcoxon signed-rank tests (*p* < 0.05).

**Table 3.** Ensemble decoding performance and ensemble size when decoding motion direction for each recording session in both animals

Recording session	Maximum BE performance (%)	BE size at maximum performance	BE size at 90% of maximum performance	Maximum BSU performance (%)	BSU size at maximum performance	BSU size at 90% of maximum performance	Total *n*
Monkey R							
Day 1	75.33	18	2	73.67	98	6	116
Day 2	67.31	30	92	62.80	79	27	94
Day 3	71.87	88	6	69.95	93	17	116
Day 4	73.36	98	7	70.26	134	23	136
Monkey S							
Day 1	91.29	33	3	90.11	95	5	112
Day 2	89.38	41	3	87.10	32	18	73
Day 3	89.51	48	14	86.61	76	49	77
Day 4	87.10	34	3	83.89	49	2	61
Day 5	87.64	42	5	87.33	66	35	68

Same as Table 2 but for the feature motion direction.

When decoding target location in monkey R (Fig. 6*E*), the best BE returned slightly higher median decoding accuracy than the best BSU (89.19% ± 3.07 and 88.00% ± 5.26, respectively), yet this difference was not statistically significant (Wilcoxon signed-rank, *p* = 0.1250^dd^). Similarly, when decoding motion direction, the best BE yielded a higher median accuracy (72.62% ± 3.42) than the best SU (70.10% ± 4.57), but this difference again did not reach statistical significance (Wilcoxon signed-rank, *p* = 0.1250^ff^).

When decoding target location in monkey S (Fig. 6*F*), the best BE generated higher median decoding accuracy than the best BSU (60.93% ± 2.05 and 57.32% ± 2.26, respectively). This difference did not reach statistical significance (Wilcoxon signed-rank, *p* = 0.0625^ee^). The same was true for the decoding of motion direction (median best BE = 89.38% ± 1.66, median best BSU = 87.10% ± 2.21; Wilcoxon rank-sum, *p* = 0.0625^gg^). In summary, when we analyzed each animal separately, we found a small trend for the median decoding accuracy to be higher when using the BE ensemble building method, but it did not reach statistical significance.

However, when pooling data across animals (*n* = 9, Tables 2 and 3, compare second and fourth columns) we found that BE method yielded significantly higher maximum performance than the BSU method for both attended location and motion direction (Wilcoxon signed-rank test, *p* = 0.0039^hh^ for target location and *p* = 0.0039^ii^ for motion direction).

### Effect of ensemble size on decoding performance

One interesting observation in Fig. 6*A–D* is that as ensembles increase in size, there is a fast increase in performance for small sizes that seems to reach an asymptote for ensembles of ∼20 units or less. This suggests that maximum classification performance can be achieved with ensembles that are substantially smaller than the largest possible ensemble size. It also suggests that the information brought about by adding more neurons to an ensemble can be negligible. To closely examine this issue, we computed the ensemble sizes at which the maximum performance was achieved, as well as the ensemble size at which 90% of that maximum performance was achieved for both the BE and the BSU methods (Tables 2 and 3).

In some ensembles, the number of neurons needed to achieve maximum performance is smaller than the number of neurons needed to achieve 90% of that performance (Table 2, monkey Sd5). This is because the best decoding performance as achieved by a single neuron, and adding more neurons lowered the performance. These ensembles were more the exception than the rule. They usually have lower performance than the rest of the ensembles, which can be explained by the progressive addition of noisy neurons that are poorly tuned and detrimental to the correlation structure.

We pooled the indices across monkeys and across direction and location ensembles (Tables 2 and 3) and compared ensemble sizes for maximum performance and 90% of maximum performance. The latter was done to have a second estimate that does not depend on a single measurement that could be due to a peak in performance at a given ensemble size. The BE method reached maximal decoding accuracy at smaller ensemble sizes than the BSU method (Wilcoxon signed-rank, *p* = 0.0198^jj^). The BE method also yielded 90% of maximum decoding accuracy at smaller ensemble sizes compared to the BSU method (Wilcoxon signed-rank, *p* = 0.0293^kk^). Overall, our results indicate that BE ensembles reach their best decoding performances at smaller ensemble sizes. This is also in line with our previously mentioned result (Fig. 6*B*), which indicated that significant differences between BE and the shuffled counterparts were found at relatively small ensemble sizes.

### Decoding accuracy and behavior

**Figure 7.**
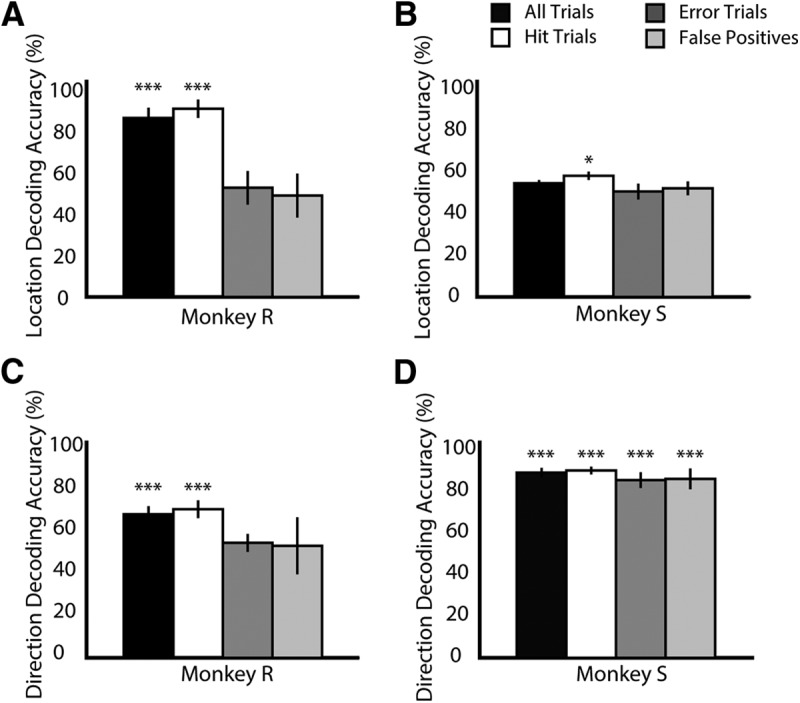
Relationship between decoding accuracy and monkeys’ behavior. We used the BE ensemble with the highest decoding accuracy to decode target location from the neuronal activity during different trial outcomes (black, averaged across all trials; white, correct trials only; dark gray, error trials; light gray, false positives only) independently for monkey R (***A***) and monkey S (***B***). Error bars represent standard deviations across recording sessions. Asterisks mark significant differences in mean accuracy compared to chance decoding accuracy (***, *p* < 0.001, *, *p* < 0.05; exact test). We repeated this analysis for motion direction in monkey R (***C***) and monkey S (***D***).

One important question is whether the information encoded by neuronal ensembles in the LPFC contributes to task performance. If it does, one would anticipate that fluctuations in ensemble performance correlate with fluctuations in the animals’ performance. We decoded the attended location using the neuronal activity from the BE ensembles that had generated the maximal decoding performance for different trial outcomes: all trials, hit trials only, relevant error trials [misses (no response) and false positives (response to change in distractor stimulus)], and false positives only. In monkey R, the average decoding performance across all trials was 83.11% (SD 4.79; Fig. 7*A*, black bar), which was significantly higher than chance decoding (exact test, *p* < 0.01^ll^) but slightly lower than the performance using hit trials only (87.40% ± 4.27, white bar; exact test to compare to chance, *p* < 0.01^nn^) whereas the decoder performed at chance level when using error trials (50.72% ± 7.80, exact test, *p* = 0.4300,^pp^ dark gray bar) or only false-positive trials (47.09% ± 10.24, exact test, *p* = 0.4600,^rr^ light gray bar).

When we decoded target location from the data obtained from monkey S, we actually did not reach higher-than-chance performance when considering all trials (52.84% ± 1.44, exact test, *p* = 0.2840^mm^; Fig. 7*B*, black bar). However, when considering hit trials only, the decoder’s performance was slightly but significantly better than chance (white bar, 56.28% ± 1.92; exact test, *p* = 0.0160^oo^). When decoding from error trials, the performance was again at chance level [48.95% ± 3. 68, exact test, *p* = 0.3760^qq^ and 50.44% ± 3.19, *p* = 0.6600^ss^ for error trials (dark gray bar) and false positives only (light gray bar), respectively].

We repeated this analysis for the feature motion direction. In monkey R, the decoder’s performance was above chance when considering all trial outcomes (Fig. 7*C*, black bar) and correct trials only (white bar; 66.68% ± 3.71 and 69.01% ± 4.15, respectively; exact test, *p* < 0.01^tt,vv^). When considering all error trials (dark gray bar), the performance dropped to chance level (53.38% ± 4.17; exact test, *p* = 0.5500^xx^). Similarly, when decoding from false-positive trials, performance was at chance level (52.01% ± 13.23; exact test, *p* = 0.3850^zz^).

In monkey S, the decoding accuracy remained high regardless of behavioral performance (Fig. 7*D*). When decoding from all trials, the mean performance was 86.03% ± 2.16 (black bar). When using hit trials only (white bar), there was a slight increase to 86.98% ± 1.79, and when using error trials (dark gray bar), it dropped to 82.52% ± 3.61, which is very similar to the decoding performance when considering false positives only (83.08% ± 4.82, light gray bar). In all cases, the decoder performed above chance level (exact test, *p* < 0.01^uu,ww,yy,aaa^).

These results indicate that in both animals there is a similar relationship between the behavior of the monkey and the encoding efficiency of LPFC neuronal ensembles, with the effect being more pronounced for the allocation of spatial attention.

## Discussion

Our data demonstrate that individual neurons and neuronal ensembles in the LPFC encode information regarding the attended spatial location and the motion direction of a stimulus. Using linear classifiers, we demonstrate that neuronal ensembles in the LPFC encode more information about these two variables than individual single units. The size and composition of the neuronal ensembles influence the amount of encoded information. Finally, our data show that the performance of neuronal ensembles at encoding information on a single-trial basis is correlated with the animals’ behavioral performance.

### Encoding of spatial attention and stimulus features in LPFC

Single neurons in the LPFC encode the location of visuospatial attention (Lennert and Martinez-Trujillo, 2011, 2013). However, how this translates into the ability of neuronal ensembles to encode the spatial location as well as nonspatial features of visual stimuli during an attentional task has not yet been extensively investigated. In our study, rhesus monkeys directed their attention to one of two moving RDPs based on a color cue and then maintained attention on the target location (spatial attention) until they detected a motion direction change in the target, ignoring any changes in the distractor.

Our data show that the encoding of attended location and stimulus motion direction (two task-relevant variables) is stronger in neuronal ensembles than in single units. This may appear to be a trivial result, since neuronal ensembles can average noise and perform better than their individual components. However, this is not necessarily true if single units have perfect classification performance (e.g., 100%). Additionally, the firing of units in the ensembles is not uncorrelated—in fact, it has been shown that in visual cortices, the correlation structure of an ensemble can impair performance and that attention reduces this effect by decorrelating neuronal firing (Cohen and Maunsell, 2009; Mitchell et al., 2009). Interestingly, in monkey S, for the encoding of the attended location, when removing correlations, the ensemble performance drops below the performance of the best single unit for some ensemble sizes (dashed lines drop below the first circular marker in the *y* axis). This agrees with a recent report of noise correlation improving the coding of working memory in the lateral prefrontal cortex (Leavitt et al., 2017). This suggests that the coding properties of LPFC neuronal ensembles and the amount of encoded information cannot simply be estimated from the information obtained from measurements of single-unit activity alone (e.g., averaging the performance of single units or choosing the maximum performance across units).

Attention is the enhanced processing of behaviorally relevant information at the expense of distractors (Treue and Trujillo, 1999; Martinez-Trujillo and Treue, 2004; Patzwahl and Treue, 2009). Spatial attention involves the allocation of attention to a relevant visual location in our environment (Posner, 1980). The effects of visual attention on neuronal responses appear to get stronger as one moves up the processing hierarchy (Treue, 2001). By the time visual signals reach the LPFC, attentional filtering is strong and arises early after a cue onset (Everling et al., 2002; Buschman and Miller, 2007; Lennert and Martinez-Trujillo, 2011; Squire et al., 2013). To guide the allocation of attention, information about visual stimuli and their behavioral relevance must be integrated somewhere in the brain. We hypothesize that area 8A of the LPFC is a likely candidate based on its anatomic and functional properties, e.g. bilateral representation of the visual field (Lennert and Martinez-Trujillo, 2011, 2013; Tremblay et al., 2015; Bullock et al., 2017), selectivity for stimulus features (Hussar and Pasternak, 2009; Mendoza-Halliday et al., 2014), and connectivity to other prefrontal areas and sensory cortices (Petrides, 2005). In our study, we are able to corroborate previous reports of an involvement of area 8A in spatial attention (Reser et al., 2013; Tremblay et al., 2015), while also demonstrating that other task-relevant parameters such as the direction of motion of a stimulus are encoded as well. Indeed, a recent report has provided evidence of a contribution of area 8A to the coding of attended nonspatial features (Bichot et al., 2015). Our study does not specifically address coding of nonspatial attention by neurons in area 8A but it does show that task-relevant stimulus features are encoded, which is likely necessary for coding of signals related to feature-based attention (such as to motion direction).

Specifically, we show that single units encode both the attended location and motion direction of stimuli. Furthermore, the populations of neurons encoding these two variables seem at least partially segregated within the LPFC, with a small proportion of units representing both types of information.

### Single-unit selectivity in LPFC

There is ample evidence that a large proportion of prefrontal neurons show task-related activity, ranging from ∼40% (Fusi et al., 2016) to almost 100% (Duncan, 2001). Specifically, many prefrontal neurons encode task-relevant parameters (Rao et al., 1997; Miller, 2013; Donahue and Lee, 2015), such as information about the stimulus properties, memory components, or reward size. These neurons may not show classic sensory neural tuning, but could selectively respond to the most relevant information for the current task (Yantis, 2008; Fusi et al., 2016).

In our study, a large proportion of neurons preferentially responded when the attended stimulus was presented in the ipsilateral or the contralateral hemifield relative to the recording site. We also found many neurons to respond more strongly to one motion direction than the opposite motion direction. In general, our results agree with previous findings of large proportions of prefrontal neurons exhibiting task-related activity and many single units having preferences for pertinent motion parameters (Hussar and Pasternak, 2009; Mendoza-Halliday et al., 2014). Although we did not explore the full range of tuning, the selectivity of our single LPFC neurons does not seem to substantially differ from the one found in visual neurons in area MT for similar parameters (e.g., motion direction).

Previous studies have reported that single units in the LPFC show mixed selectivity (Rigotti et al., 2013; Fusi et al., 2016). This is thought to increase the computational power of neuronal ensembles (Miller, 2013) by increasing the dimensionality of the neural representations. We did not find a substantial number of units selective for both attended location and the stimuli’s motion direction, the relevant parameters in our task. One possibility for this discrepancy is that in we did not employ a large enough number of parameters and our task may not have been complex enough in that respect. Furthermore, our animals were extensively trained in the task, which may have biased the selectivity of neurons toward the relevant task variables. In favor of this hypothesis, we found that the differences in selectivity between the recorded populations in the two animals correspond to their training history. The latter also suggests that although LPFC neuronal representations seem to be flexible, prolonged exposure to certain tasks and stimuli may leave a permanent “blueprint” in those selectivities. This may be the basis for specialization after prolonged training. However, this issue needs further investigation.

### Coding properties of single units versus neuronal ensembles

One interesting result of our study is that we are able to decode certain stimulus features even when the majority of single units showed no selectivity for such features. Specifically, in monkey S, the proportion of units selective for the attended location was not different from chance, yet we were able to decode the attended location from neuronal ensembles above chance level (albeit not statistically significantly higher). One likely explanation is that a small proportion of selective single units is driving the classifier’s performance. Classifiers do not represent the mean performance of a population of single units, or the maximum across units (Averbeck et al., 2006; Tremblay et al., 2015). They provide a linear estimate of the maximum amount of information encoded by the population of “simultaneously active” neurons. For the case of binary classifiers, this linear estimate is a function of the differences between the mean firing rate between conditions (or center of the cluster in a multidimensional space where each unit represents one axis) and the correlation structure of the ensemble of simultaneously active units. This second variable is complex, and it is difficult to estimate its contribution in populations of neurons with correlated firing (Moreno-Bote et al., 2014; Arandia-Romero et al., 2016; Leavitt et al., 2017). Importantly, our results show that the exact amount of encoded information about stimulus features cannot be merely deduced from individual single units encoding properties.

We found that the best ensemble (BE) method yielded better decoding accuracy than the best single unit ensemble (BSU) method for small ensemble sizes. The support vector machine (SVM) was more accurate when we did not constrain the ensembles to be composed of the best-performing single units (BSU method). This result can be easily explained if one considers that classifiers can use information regarding the correlation structure of an ensemble. So, a given unit may be highly selective for a given feature (e.g., attended location), but when added to the ensemble it may shape the correlation structure in a manner that deteriorates the classifier’s performance (Moreno-Bote et al., 2014). On the other hand, a unit that is less selective for the same feature may shape the correlation structure in a different direction and positively contribute to the classifier’s performance (Averbeck et al., 2006; Leavitt et al., 2017). This may explain why in the example sessions shown in Fig. 5 the BE method adds units to an ensemble that are not the most selective based on their individual classification performance to maximize ensemble performance.

Last, we would like emphasize that the BE method uses the best units in the population taking into account their signal and noise correlation structures. From this point of view, it does not reflect the computations performed by ensembles of average units in the population, but by ensembles of units that maximize the performance of the classifier. Whether the brain performs computations using average units or the best performing units is unclear. Thus, our results must be interpreted taking this into account.

In general, these results illustrate that information coding by neuronal ensembles is complex and cannot be solely derived from the tuning properties of single neurons.

### Effect of training history on information coding by LPFC ensembles

We found that although there were units selective for the location of spatial attention in monkey S, the proportion was not significantly different from chance level. In fact, when examining the data from both animals, we found an inverse relationship between the proportions of units selective for the attended location relative to the proportions of units selective for the motion direction. One possible explanation for this result is that the animals’ training history shaped the neuronal selectivities: monkey S had been extensively trained in various tasks involving motion direction as the relevant stimulus feature (e.g., Mendoza-Halliday et al., 2014), whereas monkey R had, at the time of recording, been exclusively trained on a spatial attention task involving a color hierarchy (Lennert and Martinez-Trujillo, 2011, 2013).

Previous studies have shown the emergence of selectivities for stimulus features or categories in lateral prefrontal cortex neurons due to training (Antzoulatos and Miller, 2011), as well as in the parietal cortex (Freedman and Assad, 2006) and the inferiortemporal cortex (Srihasam et al., 2012). Similarly, it has been suggested that training and the expertise derived from it can lead to increased tuning of neurons for certain objects (Riesenhuber and Poggio, 2002). Our results are in agreement with these reports, corroborating that neuronal selectivities in the LPFC are shaped by experience.

Our results are unlikely due to systematic behavioral idiosyncrasies (e.g., biases in response patterns) in monkey S. It is possible that this animal tried convoluted strategies to perform the task (e.g., always responding to the first motion direction change if green was on the left versus always responding to the change in the red stimulus), and even that its strategies changed from day to day. However, such strategies would have likely resulted in chance performances.

Another possibility is differences in the anatomic locations of the array implants. It is possible that in monkey S we did not record from the LPFC regions with high proportion of neurons selective for the attended location, but from regions where neurons were predominantly selective for the attended direction. In monkey R, the inverse may be true. An analysis examining whether location- and feature-selective units are significantly organized or clustered across the array suggested that there was no topographical organization of selectivity in either animal (Fig. 3*E*,*F*). However, it is possible that the arrays were implanted in different regions with different proportions of units selective for our task variables. Indeed, a recent study has shown a degree of topographical organization for visual signals and saccadic eye movements in area 8A (Bullock et al., 2017). Although we tend to favor the experience/training-dependent explanation, we cannot fully rule out the latter explanation with our dataset and the lack of knowledge of this area’s topographical organization.

One question arising from these results is whether and how the proportion of selective neurons in LPFC area 8A could change with training, as well as how long is needed for this to happen. Unfortunately, microelectrode array recordings are not identical from session to session; therefore we could not track most neurons over different sessions. However, we did not find the proportion of selectivities to change dramatically over recording sessions. One possibility is that the electrodes penetrate the surface orthogonally, and thus parallel to cortical columns; so for a single electrode, even if the depth position and therefore the recorded neurons vary from day to day, the tuning of the neurons likely remains the same.

In summary, LPFC neuronal ensembles encode attended spatial locations as well as nonspatial visual features (motion direction) with significantly higher accuracy than individual neurons. This result suggests that the LPFC contains activity maps of these two variables that can be read out by downstream or upstream areas; it also highlights that the information contained in such maps cannot be inferred from the selectivity of individual units alone. Overall, our results emphasize the importance of simultaneous measurements of neural activity in behaving animals.

## Synthesis

Reviewing Editor: Tatiana Pasternak, University of Rochester Decisions are customarily a result of the Reviewing Editor and the peer reviewers coming together and discussing their recommendations until a consensus is reached. When revisions are invited, a fact-based synthesis statement explaining their decision and outlining what is needed to prepare a revision will be listed below. The following reviewer(s) agreed to reveal their identity: Klaus Wimmer, Tatiana Engel

Both reviewers had a number of positive comments about the manuscript but also had a number of serious reservations that must be addressed before the paper can be accepted for publication in eNeuro.

These are summarized below:

1. Both reviewers question whether the behavioral task used in the study actually probes feature-based attention, in addition to spatial attention. The revision and the response to the reviews must address this important point.

2. Information about the correlation structure in the ensembles should be provided and included in the discussion of the results (point raised by Rev2 and supported by Rev1). This analysis will determine whether the superiority of neuronal ensembles is due to the correlation structure, the feature not considered in decoding of single units.

3. Training history vs placement of recording arrays (Comment 2 from Reviewer 2). This is a valid issue, considering that the differences between the two animals include both training history and recording locations. Incidentally, there appears to be a mistake in the diagram in Fig 2 showing the relative location of the arcuate and the principal sulci (the distance between the two in monkey R and Monkey S).

4. Please address all the ‘Minor Comments’ provided by both reviewers.

REVIEWER 1.

This paper investigates how ensembles of PFC neurons represent information about visual motion stimuli, and how this representation depends on the task demands (i.e. attentional condition). The paper is well written and in general the experiments and data analysis stand on solid grounds. I am confident that the manuscript is suitable for publication in a journal such as eNeuro. My main concerns are related to the interpretation of the results in the light of spatial and feature-based attention (see below) which I think can be dealt with in a revised version.

Major:

Feature-based and spatial attention: The authors use a spatial attention task with two simultaneously presented RDPs, one of which is cued. In each trial, the RDPs move in one of two possible directions and the task of the monkey is to report a direction change in the cued RDP while ignoring a change in the distractor stimulus. This experimental design does not allow to say anything about ‘feature-based’ attention. It is probing spatial attention, in fact very similar to Exp 1 in Treue & Martinez-Trujillo, 1999. In the experiment presented here, the motion direction of both stimuli is always identical, so how could attention be directed selectively to one direction? My suggestion is to rewrite the paper in order to remove the idea that feature-based attention to the direction of the RDPs is tested with this task. In particular, the design does not allow to discern the encoding of stimulus direction vs. feature-based attentional modulation (as suggested in the introduction, line 68ff). I think that statements such as in line 254f are misleading: “Within a trial the animals attended either to the right or left RDP (spatial attention) and to either up or down motion (feature-based attention”.

Minor:

1. Monkey S does not hold fixation very well (4 deg fixation window). Is 4 deg the diameter or the radius of this window? Monkey S has participated in different experiments previously. Did he also have relatively poor performance in these tasks and might this be related to poor fixation?

2. Line 175: What does it mean that the ‘firing rates were shuffled’? I thought the trial labels would be shuffled between different conditions (between the four conditions: 2 spatial and 2 different directions).

3. In general, I would suggest to use >=1000 repetitions for permutation tests (rather than 100 as done here).

4. Line 185/186: this does not describe 10-fold cross-validation but some sort of Monte Carlo cross-validation. In 10-fold CV, each trial would be exactly once in the test set and 9 times in the training set.

5. Fig 3A: the numbers provided below the Venn diagram seem to be more informative than the Venn diagram itself. Perhaps bar plots with whiskers showing the s.e.m. would be an alternative?

6. Line 320: what is T_i and T_j ?

7. Line 333: I don't see that it is shown that both animals had a response modulation depending on the attended location. In monkey S, there was no significant effect for individual neurons (in Fig 2A). In Fig 4B neurons are aligned depending on their ‘preferred location’. How was this done exactly? I suspect that the red and blue lines diverge just because of this alignment and not because there is a true effect. In order to determine the statistical significance of the difference in Fig 1B, surrogate data (shuffles) could be used (as in Fig 6B).

8. Fig. 6B, text line 406: I wonder if the Nmax decoding accuracy is still above chance level?

9. In the same figure, why is the Nmax performance not identical for BE and BSU (concerns all panels in Fig. 6)? Shouldn't it be equal by definition?

10. Related, why is the performance for hit trials in Fig 7B not significantly different from chance? This seems to be different than in Fig. 6B?!

Discussion starting line 592: I agree that is interesting that stimulus features can be decoded more reliably from the ensemble than from single units but I am not sure if this is supported by the data of monkey S (Fig. 7B).

REVIEWER 2.

The paper examines how spatial and feature attention signals are represented by single neurons and by neuronal ensembles recorded simultaneously in the LPFC. Selectivity of single neurons is quantified using analysis of variance, and selectivity of neuronal ensembles is quantified using a linear classifier performance. The conclusion is reached that neuronal ensembles represent information more efficiently than individual single neurons. The paper is well written, and the data is presented clearly. However, in my opinion, the presented analyses are not sufficient to defend the authors' conclusion that neuronal ensembles encode information more efficiently than individual neurons. There are three main points that need to be addressed:

1) The conclusion that ensembles are more efficient than single neurons is based on the observation that (i) the ensembles consistently yield higher decoding accuracy than the best single units (e.g., lines 381-382); and (ii) classifiers built using the best ensemble (BE) method perform better than classifiers built using the best single unit (BSU) method for intermediate ensemble sizes. However, the observation (i) is trivial because even when we are dealing with an ensemble of noisy uncorrelated units, the ensemble will outperform the best unit simply due to noise averaging in the pooled population response. As for the point (ii), it is argued that it indicates that there is some interesting correlation structure in the ensembles which makes BE outperform BSU. However, this claim is not supported by any analyses. First, it seems that the BE method is designed to perform at least not worse than the BSU method. I tried to ran the two methods on synthetic data generated as ensembles of uncorrelated noisy units, and observed that the BE method consistently outperforms the BSU method although there was no structure in this synthetic ensembles beyond what is given by individual units. So, is the observed improvement of the BE over BSU greater than what is expected by the methods design? Can the beyond-expected improvement be quantified using synthetic data or manipulations of the real data? Second, why is the improvement of the BE over BSU attributed to some correlation structure in the ensembles rather than to relative arrangements of the mean responses in the high-dimensional space? For example, if within the same condition trials are shuffled to destroy all correlations between the units in the ensemble (but mean and variance are unchanged), will then BE method perform the same as BSU? Without answering these questions, it seems, we can't say for sure that ensembles encode something beyond just a combination of individual units.

2) Differences between the two animals in neuronal selectivities for the attended location and attended feature are attributed to differences in the animals' training histories. However, this claim is not sufficiently supported by the data. The only data presented for this issue is in Figure 3B, showing training histories of the two animals. However, to argue that tuning differences arise from the different training histories it is necessary to show how neuronal tuning is changing over the course of training on a particular task. Over the course of recording sessions (which is a relatively short time period), no clear changes in tuning has been observed (Figure 3C). Therefore, it remains unclear whether and how training history affects neuronal selectivity in the LPFC. The alternative and equally viable hypothesis is that tuning differences result from different placement of the recording arrays in two animals and topographical segregation of location- and feature-selective units within the LPFC. This alternative hypothesis is briefly mentioned in Discussion, but it is given much less weight and consideration relative to the training-history hypothesis, which seems unjustified. To find some support (or the lack of thereof) for the feature-topography hypothesis, it is possible, for example, to examine how the location- and feature- selective units are organized in space across the array. Did the units with the same selectivity tend to cluster? How did this change across sessions? There is one sentence which seems to refer to something like this (lines 309-311), but unfortunately it is a very unclear sentence, and I couldn't figure out what it means.

3) From the task description, it is not clear to me, what is feature attention in this task. It seems, that animals were cued using stimulus color to attend to one location vs. the other. Was the motion direction in the two stimuli always different, or was it the same on some trials (as is shown in Figure 1A)? If the motion direction is the same for both stimuli, then what is the attended feature? This needs to be clarified.

Minor points:

1) lines 152-154: was the window centered around the stimulus onset? Why was this window selected? How early did different neurons showed attentional modulation?

2) lines 162-163: I suppose this is referring to trial-average responses, but this is not stated. Please clarify.

3) line 179: what was the regularization parameter? How was it chose? How the number of trials was balanced across conditions?

4) lines 309-311: not sure what this sentence means.

5) line 380: typo: performing/unturned should read performing/untuned.

6) lines 402-404: this might also be trivially a result of noise averaging in an ensemble of uncorrelated units.

7) lines 406-407: unclear, how small trial number leads to performance decline. Please explain.

8) lines 420-424: again, this may be a trivial result of noise averaging.

9) lines 428-430: unclear, what is the maximum performance and why small number of trails is important here (what does this explain?). Unclear.

10) Figure 6A-D. Not sure what the right ordinate axes are for? The numbers are the same as on the left ordinate axes.
